# Measuring Chuckwagon Racehorse Movement Asymmetry Before and After Racing Using Wearable GNSS-IMUs: A Preliminary Study

**DOI:** 10.3390/ani16091361

**Published:** 2026-04-29

**Authors:** Camille M. Eamon, Matthijs van den Broek, Karelhia Garcia-Alamo, Charlotte De Bruyne, Brittany L. Davis, Maggie Fallscheer, Sara Frostad, Ed Pajor, Sara Skotarek Loch, Renate Weller, Zoe Y. S. Chan, Thilo Pfau

**Affiliations:** 1 Faculty of Kinesiology, University of Calgary, 2500 University Drive NW, Calgary, AB T2N 1N4, Canada; 2 Faculty of Mechanical Engineering, Delft University of Technology, Mekelweg 2, 2628 CD Delft, The Netherlands; 3 Faculty of Veterinary Medicine, University of Calgary, 2500 University Drive NW, Calgary, AB T2N 1N4, Canada

**Keywords:** chuckwagon racing, movement symmetry, MSK injury, wearable sensors, IMUs, pre- and post-racing, racehorse, locomotion

## Abstract

In Chuckwagon racing, teams of four Thoroughbred horses pull wagons at high speeds. Movement symmetry is a key locomotion metric linked to force production, racing direction, and lameness. Racehorse symmetry in trot during on-track warmups and cooldowns was assessed. Over 10 days, 60 horses (average 8 per day) were fitted with Global Navigation Satellite Systems combined with Inertial Measurement Unit (GNSS-IMU) sensors. Weight-bearing asymmetry was quantified using the minimum difference (MnD) in vertical trunk displacement between diagonal limb pairs, and push-off asymmetry was quantified using the upwards difference (UpD). Absolute (mm) and normalized (% ROM) asymmetries were compared between warmups and cooldowns using linear mixed models. Mean MnD was similar between warmup (6.2 mm; 17.6%) and cooldown (6.4 mm, 19.7%). Mean UpD increased from warmup (11.3 mm, 31.7%) to cooldown (12.8 mm, 38.0%), with UpD% significantly higher in cooldown (*p* = 0.046). No other differences were significant (all *p* ≥ 0.202). One horse sustained a catastrophic musculoskeletal (MSK) injury. This horse’s UpD ranged from 3.3–29.7 mm (11.4–69.3%) during warmups and 24.3–25.5 mm (47.8–76.4%) during cooldowns. Push-off asymmetry may increase after Chuckwagon racing. The injured horse showed high asymmetries, but high values also occurred in uninjured horses. Further work needs to establish normal asymmetry ranges in Chuckwagon racing and identify patterns associated with MSK injuries.

## Simple Summary

Chuckwagon racing is a sport where teams of four horses pull a wagon around a racetrack. Movement symmetry, or the difference between left and right limb movement, is commonly used as an indicator of abnormal gait in horses. Symmetry is measured at the trot, a normally symmetrical gait, but it is unknown whether Chuckwagon racehorses have distinct asymmetries given their unique racing style. In this study, we used wearable sensors to measure movement symmetry as Chuckwagon racehorses trotted before and after competitive races. Horses were fitted with sensors on their harnesses, and sensors recorded during standard warmup and cooldown exercises on the track. We found that Chuckwagon racehorses may become more asymmetrical in the way they push off the ground after a race when measurements are normalized to stride range of motion (ROM). One horse with high push-off asymmetries sustained a fracture during racing, but other horses with higher asymmetries did not. Future work should establish normal ranges of asymmetry before and after competitive Chuckwagon races and assess their relation to musculoskeletal (MSK) injuries in Chuckwagon racing.

## 1. Introduction

Chuckwagon racing is a popular form of horseracing in Alberta, Canada and is rooted in many rural communities and traditions [1]. Unlike other types of Thoroughbred racing where individual horses are mounted by jockeys, Chuckwagon racing is performed by teams, otherwise known as outfits. Four Thoroughbreds are harnessed together and to the wagon, which must weigh a minimum of 600 kg with the driver in the seat. Each horse has a position as left leader (LL, front left horse), right leader (RL, front right horse), left wheeler (LW, positioned behind the left leader and in front of the wagon), or right wheeler (RW, positioned behind the right leader and in front of the wagon). Before racing around the typically five-furlong or half-mile track, the outfits perform a unique figure-eight maneuver around two barrels in the infield [1]. The combination of tight turns while rapidly accelerating followed by a high-speed gallop is not seen in other types of Thoroughbred racing such as flat racing. This raises the question of whether the locomotor characteristics of these horses differ from those of other Thoroughbred racehorses.

The locomotion of racehorses is of particular interest as certain movement patterns can give insights to musculoskeletal (MSK) stress leading to an injury. Many catastrophic racehorse MSK injuries (injuries resulting in death) represent an accumulation of MSK overloading over time, and are more chronic than acute in nature [2]. Importantly, MSK injuries are the most common cause of racehorse death [3,4,5], making them a substantial risk for researchers and stakeholders to manage in order to promote the welfare of racehorses [6,7]. However, there are no peer-reviewed, published statistics available for MSK injury risk specific to Chuckwagon racehorses. Given that distinct populations of racehorses performing different racing styles have unique movement patterns, it is unclear how closely matched Chuckwagon racehorse locomotor characteristics are to other disciplines. Before MSK injury prediction models can be investigated in this unique discipline, a baseline of locomotor characteristics must be established. We have previously shown that Chuckwagon racehorses have a significantly shorter stride length and higher stride frequency in curved sections of a racetrack compared to the straight during training sessions [8]. However, the movement of Chuckwagon racehorses has not been extensively studied and it is unknown how their movement symmetry compares with other disciplines.

Asymmetrical movement is used as a key indicator of lameness by veterinarians [9]. Horses are commonly visually evaluated at the trot, a normally symmetrical gait [10,11]. While visual assessments remain a key portion of lameness evaluations, gait analysis tools such as wearable Inertial Measurement Units (IMUs) have become increasingly popular to monitor movement symmetry more objectively and regularly [12,13,14,15]. Using practical technologies, studies have shown that racing Thoroughbreds commonly have asymmetries that can vary over days or weeks [16,17,18], and that these asymmetries are associated with consistently training and racing in one direction [17]. While Chuckwagon racing is unique from other types of Thoroughbred racing, it is unclear whether Chuckwagon racehorses have a normal range of asymmetry that is different from previously reported values [19]. A better understanding of movement symmetry patterns in Chuckwagon racehorses may provide a baseline for future longitudinal work aiming to develop injury prediction models for this unique population.

While teams of four Chuckwagon racehorses pull the wagon together, individuals move at a self-selected gait, often trotting or less frequently cantering, during warmups and cooldowns on the track directly before and after each race. Wearable sensors allow for symmetry assessment while horses are trotting on the track during these exercises without any disruption to current racing practices. Global Navigation Satellite Systems combined with IMU (GNSS-IMU) devices have been previously used for measuring stride characteristics in racehorses, making them practical for use in racing settings [20,21]. We conducted a preliminary study in which we used GNSS-IMU technology to measure movement symmetry before and after competitive Chuckwagon races. We hypothesized that Chuckwagon horses would show increased trot movement asymmetries in cooldowns compared to warmup periods, reflecting the accumulation of MSK stress over the racing period or fatigue after racing.

## 2. Materials and Methods

### 2.1. Horses and Sensor Instrumentation

We conducted a preliminary study in which 60 racehorses were convenience sampled from 11 participating outfits throughout 10 days of racing at the 2025 Calgary Stampede Rangeland Derby. A total of 10 GNSS-IMU (Racebox, Racelogic, Novi, MI, USA) sensors were used, with one sensor fitted on the harness of one horse per participating outfit every day. On average, out of the 108 Chuckwagon horses racing across the 9 race heats every day, there were 8 participating horses per day (range: 4–10), and 6 different horses per outfit (range: 1–8) over the entire period.

**Figure 1:**
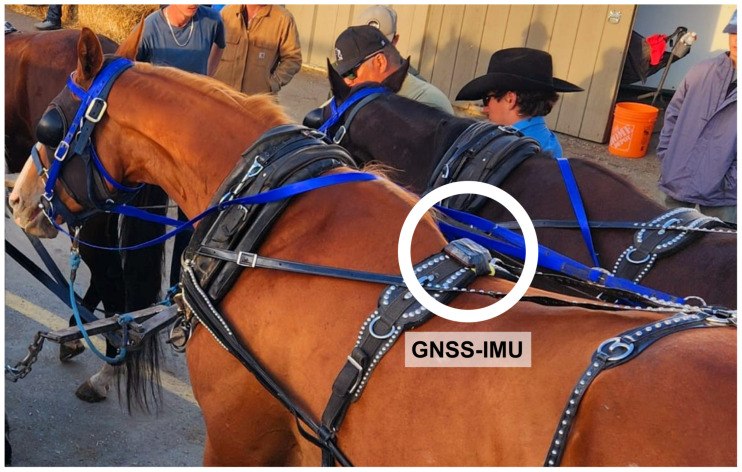
Placement of GNSS-IMU sensors on the harnesses of Chuckwagon racehorses. Sensors were secured prior to horses being harnessed to the wagon for minimal disruption to routine racing practices implemented by each outfit.

For each horse, a median of 2 (range: 1–7) measures were obtained. Calgary Stampede Fitness to Compete regulations require that Chuckwagon racehorses running for three consecutive days receive one day of rest, and after four consecutive race days they must be given two rest days [22]. Drivers chose which horse from their outfit would wear the sensor each day. This led to a different number of measures for any given horse depending on how often they participated and if they self-selected a trotting gait (during warmup and/or cooldown) on any given day. GNSS-IMU sensors sampling GNSS (speed, longitude, latitude, and altitude) and IMU (tri-axial acceleration and tri-axial rate of turn) at 25 Hz were placed on the harness at the midline in the cranial/mid thoracic area (Figure 1).

Each day prior to racing, sensors were secured to the corresponding harnesses of participating horses. Horses were then harnessed, hooked together, and attached to the wagon before their race. Participating horses could be in any of the four standard Chuckwagon positions, left leader (LL), right leader (RL), left wheeler (LW), or right wheeler (RW) on an outfit. The distribution of horses recruited was balanced to achieve a similar number of left and right leaders and wheelers evaluated and typically restricted to only one horse per outfit to minimize potential disruption and interference with routine racing practices. Of the horses in the sample, 15 were used as LLs, 18 were RLs, 13 were LWs, and 15 were RWs (total of 61; one horse was a RW on one day and a LW on another day).

The procedures of the current study were approved by the University of Calgary Animal Care Committee (Approval number: AC23-0010), and written consent from the owners was obtained prior to testing. Drivers must record their team to compete each day into a software program before 3 pm (four hours before the Rangeland Derby begins), so that official race veterinarians can evaluate the health and soundness of each horse running prior to race time. This is an important aspect of the Fitness to Compete program at the Calgary Stampede [22]. All the Chuckwagon horses competing must be microchipped, and scanners are used to positively identify each horse both at the pre-race veterinary evaluation, as well as before all the horses enter the track [22].

### 2.2. Racing Protocol

There were 1620 race starts total over 10 days of consecutive racing. Each heat had 3 outfits competing against each other. Outfits consisted of 4 wagon horses and 2 outrider horses (additional mounted horses that follow the wagons), for a total of 6 horses per outfit, or 18 horses per heat. With 9 heats in a day, this constituted 162 race starts (108 wagon horses and 54 outrider horses) per day, for 1620 race starts total. The sensors were distributed across the 9 heats according to the racing schedule of participating outfits, with at least one sensor in a median of 7 heats per day (range: 4–8).

**Figure 2:**
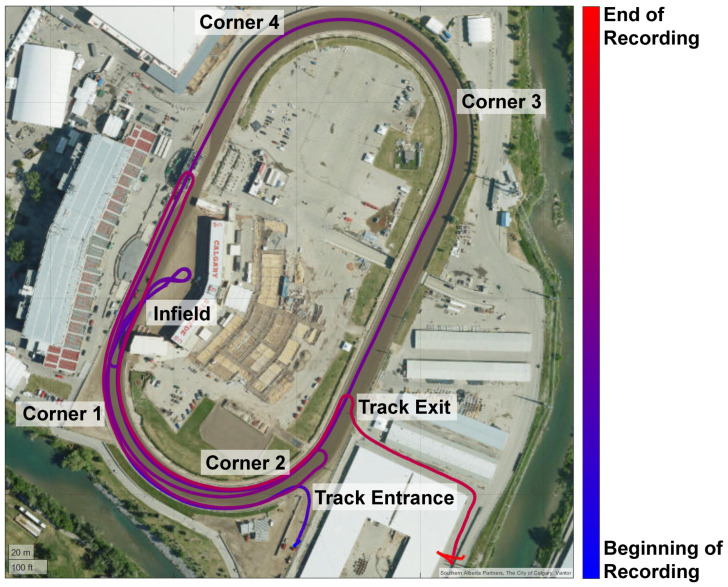
Schematic demonstrating a recording during warmup (south curve of the track and practice figure-eight maneuver), throughout racing around the track, and cooldown (past the grandstand, south curve of the track, and exiting the track along the back straight). The blue colour represents earlier in the recording, while red represents later in the recording. Tracing was obtained using Matlab’s Geoplot function.

Sensors were activated remotely using the Racebox app from multiple smartphones, including iOS and Android devices, as outfits waited to enter the racetrack at the Track Entrance (Figure 2). The outfits lined up there before the race to have their microchips scanned to verify the identity of each horse before each race [22]. Recording was active throughout the entire time horses were on the track including warmup, racing, and cooldown. In Figure 2, the blue colour represents earlier in the recording, while red represents later in the recording.

For each race, warmup exercises began as outfits entered the track. They began by travelling along the south end of the track past Corner 2 and Corner 1 to arrive at the starting line on the infield, where they performed a practice figure-eight maneuver around the two starting barrels. They then re-positioned themselves at the starting barrels, concluding the warmup exercise. Racing consisted of a second figure-eight maneuver rapidly accelerating, followed by completing a lap around the track, and crossing the finish line in the infield. Cooldown exercises began with outfits using the south bend of the track to turn around and return to the infield, loop around the infield, and then exiting the track along the back straight (Track Exit, Figure 2). Cooldown exercises were considered completed as the horses exited the track.

### 2.3. Stride Parameters

**Figure 3:**
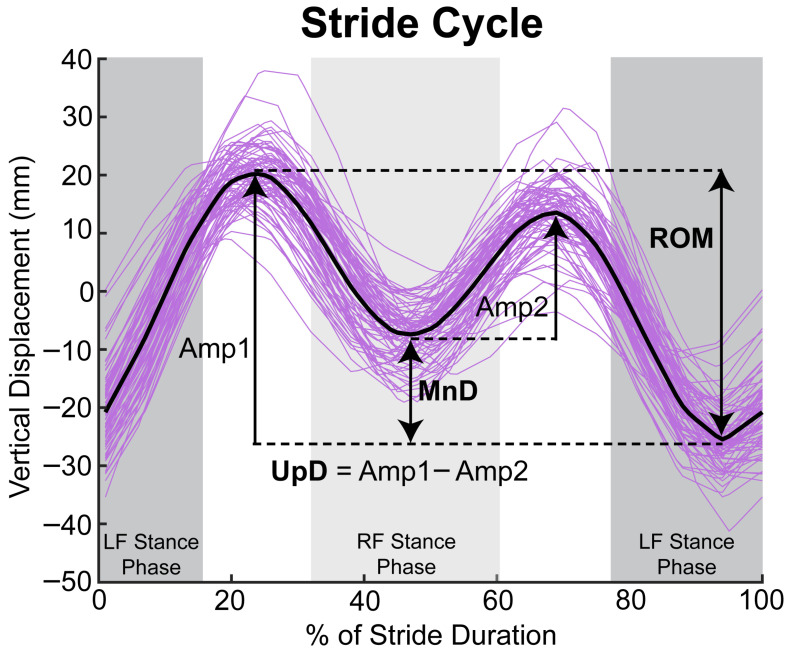
Example traces of vertical trunk displacement throughout stride cycles used to calculate MnD, UpD, and ROM. MnD was calculated as the difference between vertical displacement minima. Amp1 and Amp2 represent the amplitudes of upward movement (from the lowest vertical position during mid stance to the highest vertical position in the aerial phase) in the stride cycle. UpD was calculated as the difference between the two amplitudes of upward movement. ROM was the entire vertical displacement of a stride cycle. LF stance phase indicates when the left front foot was in contact with the ground, and RF stance phase indicates when the right front was in contact with the ground.

Trotting segments were identified using speed and vertical acceleration plots where lower acceleration amplitude combined with higher frequency identified trotting compared to cantering at comparable speed. Published algorithms [20] were used to calculate stride-by-stride vertical displacement minimum difference (MnD—which reflects weight-bearing asymmetry), upward difference (UpD—which reflects push-off asymmetry), and the entire vertical displacement of a stride cycle, referred to as range of motion (ROM) (Figure 3). MnD, UpD, and ROM values were obtained in absolute units (mm without ± signs) to prevent opposing deviations from cancelling each other out. Additionally, MnD and UpD values were also expressed relative to the ROM to obtain normalized measures (% ROM).

### 2.4. Statistics

Linear mixed models were performed using custom scripts and the fitlme function in MATLAB 2023a (Mathworks, Natick, MA, USA) to assess whether movement asymmetry significantly changes between warmup and cooldown for a competitive Chuckwagon race. Mixed models can handle uneven repeated measurements [23], and fitlme specifically uses restricted maximum likelihood estimation to help handle uneven observations per individual, making this model appropriate for our design. We compared movement symmetry for absolute (mm without ± sign) and normalized (% ROM) MnD and UpD between warmup and cooldown (fixed factor) with horse used as the random factor (*p* < 0.05). The same models were used to compare ROM between warmup and cooldown. Models were visually inspected for normal distribution of residuals. Beta coefficients, standardized effect sizes (Cohen’s d), 95% confidence intervals, and percentages of variance attributed to between-horse and within-horse differences are reported in the results.

### 2.5. Injury Data

Measuring injuries was not an objective of this preliminary study, but one horse in our sample happened to sustain a catastrophic injury while wearing a sensor during a race on the 9th day of racing. The injury was a catastrophic left forelimb cannon bone fracture, categorized as an MSK injury resulting in death. Drivers had the choice of which horses participated on any given day, so by chance this horse was instrumented with a sensor on four different occasions. Across a period of 5 days, we obtained measures for this horse 4 days, 3 days, and 1 day before the injury as well as on the day the injury occurred. Consequently, we obtained 4 warmup measures and 3 cooldown measures for this horse (no cooldown measure on the day of the injury). While the data cannot be used to conclusively assess the relationship between asymmetries and injuries in Chuckwagon racehorses, it was opportunistically collected and has been included here in the interest of beginning to move towards injury research in this unique discipline going forward.

## 3. Results

Data were obtained from 60 participating horses completing warmup and cooldown exercises over the 10 days. From these warmups and cooldowns, we collected as many clear, consecutive trot strides as possible for each warmup and cooldown. A total of 156 (73 warmup, 83 cooldown) valid trot segments were obtained. A given trot segment included an average of 38 strides (range: 13–62) and an average speed of 4.20 m/s during warmup and 4.19 m/s during cooldown. Median MnD, UpD, and ROM were calculated for each trot segment from each horse. Table 1 displays the breakdown of these trot measures over the 10 days.

### 3.1. Push-Off Asymmetry May Increase After Racing

**Figure 4:**
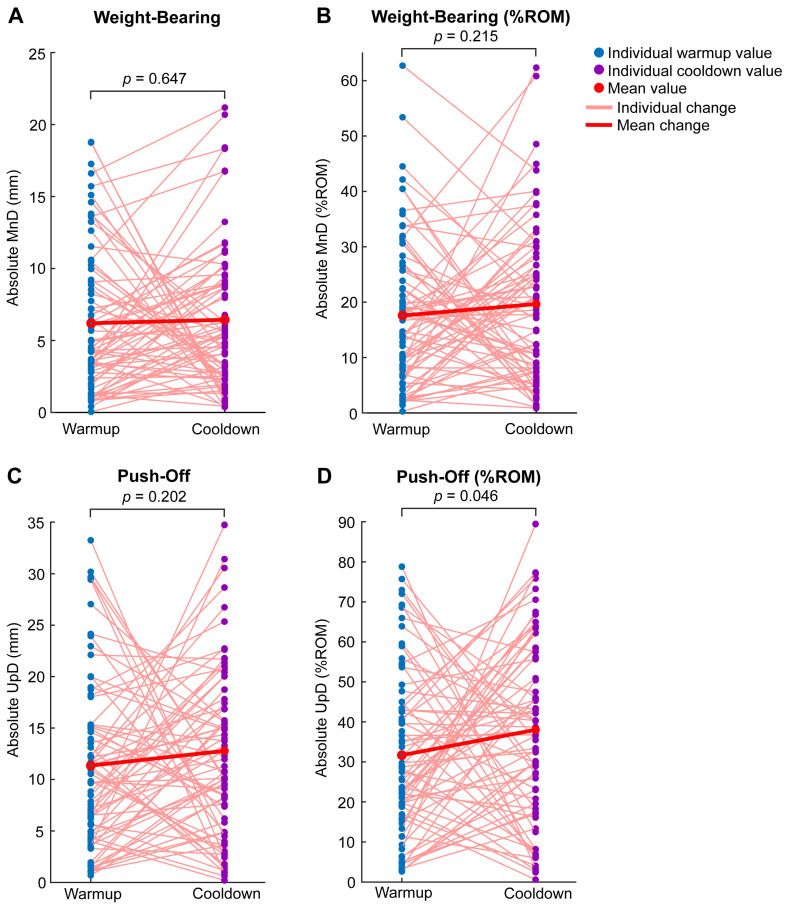
Spaghetti plots demonstrating absolute and % ROM MnD (**A**,**B**) and UpD (**C**,**D**) calculated from vertical trunk displacement quantified with GNSS-IMU sensors attached to Chuckwagon horse harnesses during warmups before racing and cooldowns after racing. Of the four parameters, UpD as % ROM was found to be statistically higher after racing. All measures obtained are shown, including those from an injured horse described in the following section. The *p*-values shown in plots are from linear mixed models performed to compare warmup and cooldown values.

**Figure 5:**
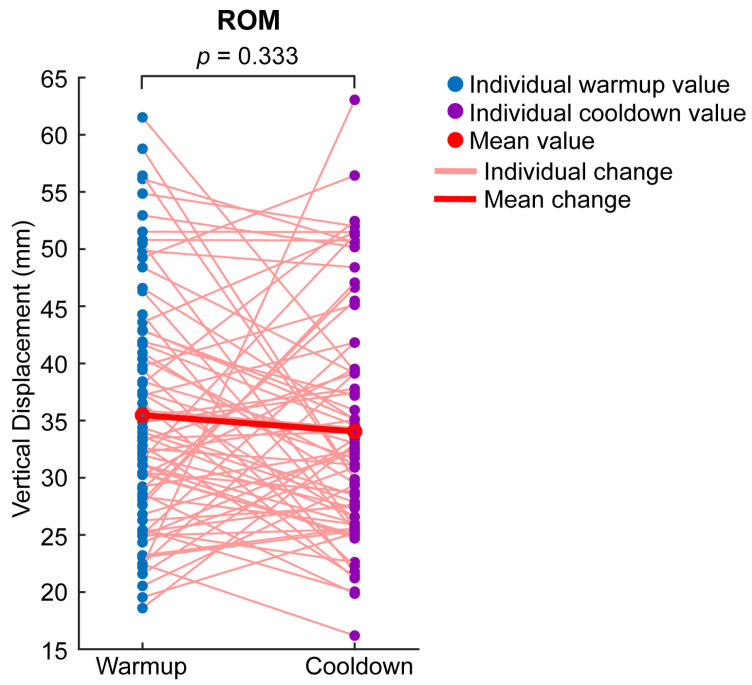
Spaghetti plot demonstrating vertical trunk ROM quantified with GNSS-IMU sensors attached to Chuckwagon horse harnesses during warmups before racing and cooldowns after racing. All measures obtained are shown, including those from an injured horse described in the following section. The *p*-values shown in the plot are from linear mixed models performed to compare pre and post values.

The average absolute MnD was 6.20 mm (17.60% ROM) during warmup and 6.44 mm (19.66% ROM) during cooldown (Figure 4A,B). The average absolute UpD was 11.35 mm (31.68% ROM) during warmup and 12.78 mm (38.02% ROM) during cooldown (Figure 4C,D). ROM averaged at 35.47 mm during warmup and 34.05 mm during cooldown (Figure 5).

Linear mixed models found a significant difference between warmup and cooldown for absolute UpD as % ROM (β = 6.55, 95% CI [0.11, 13.00], *p* = 0.046) (Figure 4D). The standardized effect size was small-to-medium (Cohen’s d = 0.32, 95% CI [0.01, 0.64]). Between-horse variability accounted for approximately 87% of the total variance for this parameter, with the remaining 13% attributed to within-horse variability. In contrast, there were no significant differences between warmup and cooldown for absolute MnD (β = 0.33, 95% CI [−1.09, 1.76], *p* = 0.647) (Figure 4A). The standardized effect size was small (Cohen’s d = 0.07, 95% CI [−0.24, 0.39]). Between-horse variability accounted for approximately 79% of the total variance here, with the remaining 21% attributed to within-horse variability. Similarly, no significant differences were observed for absolute MnD as % ROM (β = 2.39, 95% CI [−1.41, 6.19], *p* = 0.215) (Figure 4B). The standardized effect size was small (Cohen’s d = 0.20, 95% CI [−0.12, 0.52]). Between-horse variability accounted for approximately 84% of the total variance in this parameter, with the remaining 16% attributable to within-horse variability. No significant differences were observed for absolute UpD (β = 1.55, 95% CI [−0.84, 3.93], *p* = 0.202) (Figure 4C). The standardized effect size was small (Cohen’s d = 0.21, 95% CI [−0.11, 0.53]). Between-horse variability accounted for approximately 83% of the total variance for this parameter, with the remaining 17% attributable to within-horse variability. Lastly, no significant differences were observed for ROM (β = −1.24, 95% CI [−3.76, 1.28], *p* = 0.333) (Figure 5). The standardized effect size was small (Cohen’s d = −0.16, 95% CI [−0.48, 0.16]). Between-horse variability accounted for approximately 58% of the total variance for this parameter, with the remaining 42% attributable to within-horse variability.

### 3.2. Elevated Asymmetry in One Horse Approaching a Fracture

**Figure 6:**
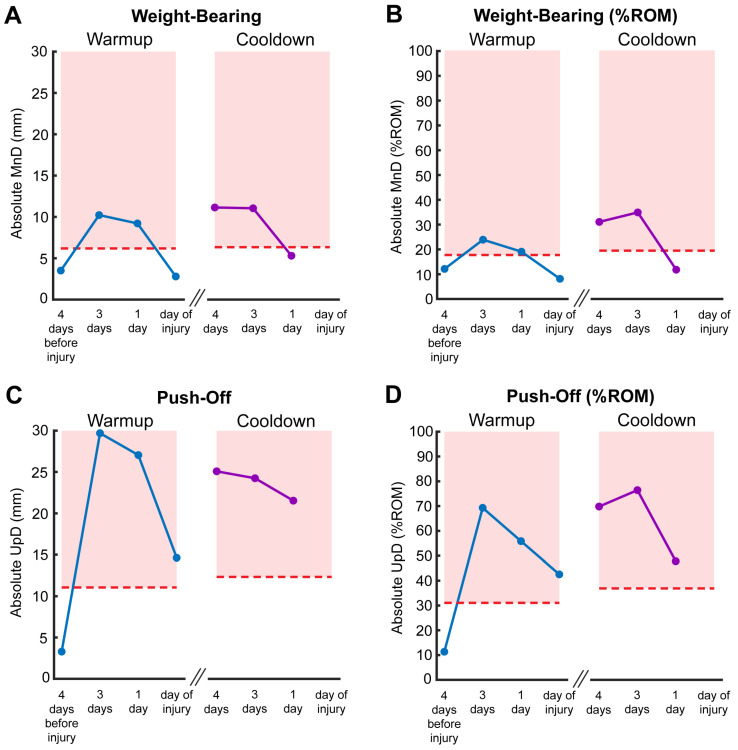
Absolute and % ROM MnD (**A**,**B**) and UpD (**C**,**D**) values across four pre-race and three post-race measures for the injured horse compared to the means of all uninjured horses in the sample. Dashed red lines indicate the means of all other horses for that given measure. Red shaded areas indicate a value above the mean of the uninjured horses.

Out of the 1620 race starts for all horses Chuckwagon racehorses competing over the 10 days of racing, one horse sustained an MSK injury resulting in death. MnD values across the four warmup measures collected for this horse were 3.51 mm (12.10% ROM), 10.22 mm (23.86% ROM), 9.22 mm (19.04% ROM), and 2.79 mm (8.13% ROM) (Figure 6A,B). MnD values across the three cooldowns were 11.14 mm (31.01% ROM), 11.05 mm (34.82% ROM), and 5.30 mm (11.77% ROM). These values are shown relative to the means of all other horses in Figure 6.

Of the 59 uninjured horses, 14 had warmup MnD values above the injured horse’s highest warmup MnD value (23.73% of uninjured horses). Eighteen had warmup % ROM MnD values above the injured horse (30.51%). For both parameters, one horse had values above the injured horse on two different days. For cooldowns, 10 uninjured horses had MnD values above the injured horse’s highest value (16.95%), and 10 horses had % ROM MnD values above the injured horse (16.95%). The same uninjured horse who had multiple warmup values above the injured horse also had two cooldown values above for MnD and three values above for % ROM MnD.

UpD values across four warmup measures for the injured horse were 3.29 mm (11.36% ROM), 29.70 mm (69.30% ROM), 27.05 mm (55.87% ROM), and 14.62 mm (42.53% ROM) (Figure 6C,D). UpD values across the three cooldowns were 25.08 mm (69.80% ROM), 24.25 mm (76.42% ROM), and 25.53 mm (47.80% ROM). With the exception of warmup measures 4 days before the MSK injury, these values are consistently elevated above the means of all other horses across all 10 days (Figure 6).

For UpD values, two uninjured horses had warmup values above the injured horse’s highest UpD value (3.39% of uninjured horses), and four had warmup % ROM UpD values above the injured horse (6.78%). For cooldowns, five uninjured horses had UpD values above the injured horse’s highest value (8.47%), and four horses had % ROM UpD values above the injured horse (6.78%).

## 4. Discussion

### 4.1. Asymmetries Observed in Chuckwagon Racing and Other Harness Racing Disciplines

In this study, we quantified Chuckwagon racehorse movement asymmetries before and after racing. At the group level, we observed average weight-bearing asymmetries of 6.20 mm (17.60% ROM) during warmup that increased non-significantly to 6.44 mm (19.66% ROM) during cooldown. Absolute push-off asymmetry also did not significantly increase from warmup to cooldown (11.35 mm to 12.78 mm). However, push-off asymmetry when normalized as percent range of motion (% ROM) did increase significantly after racing (from 31.68% ROM to 38.02% ROM).

Regarding this significant increase, absolute push-off asymmetry values (the numerator of the normalized value) were not significantly increased, nor was range of motion (the denominator) significantly decreased. Thus, it appears that the combination of non-significantly increased push-off asymmetries and non-significantly decreased ROM may explain why push-off asymmetry as % ROM significantly increases after racing in our sample. This also suggests that normalization may be necessary to detect differences. Increases in push-off asymmetries may not be solely driven by changes in movement amplitude, but rather reflect individual, limb-specific differences in push-off relative to available motion after racing.

Perhaps the need to pull the load of the wagon while attached to three other horses has a bigger influence on how Chuckwagon horses push off the ground compared to their load-bearing capacity. Similarly in Standardbred racing, individual horses pull a two-wheeled cart and driver. Johnston et al. (1999) found that pulling a horizontal load changed stride characteristics, such as reducing stride length, in Standardbred racehorses [24]. Another study by Aarts et al. in 2025 found that Standardbred trotters had reduced vertical head and withers ROM after exercise testing with a cart, which may be indicative of gait alterations due to fatigue [25]. We did not find that vertical trunk ROM significantly decreased after racing in our sample. This raises the question of whether individual Chuckwagon racehorses are limited in their ability to use compensatory mechanisms because they are influenced by the horses around them. If that is the case, it is possible that individual Chuckwagon racehorses may not express the same movement asymmetry patterns that researchers and clinicians use to quantify lameness in other disciplines.

A study conducted by Bell et al. in 2016 compared IMU measures of pelvic movement symmetry with ground reaction forces in various breeds [26]. They found push-off asymmetry was associated with a transfer of vertical to horizontal ground reaction impulse in the second half of the stance where forwards thrust is created. Taken together with the findings by Johnston et al. (1999) [24] and Aarts et al. (2025) [25], there may be a relationship between the forces required to pull a wagon and the asymmetries expressed by Chuckwagon racehorses. We were unable to assess pelvic movement symmetry as we only had a limited number of sensors available for this preliminary study, alongside the necessity of attaching equipment during racing with minimal interference to routine racing practices. Our previous work indicates that placing the IMU in the area of the harness used in the present study provides accurate locomotion data [8]. If feasible without interfering with racing equipment, having multiple IMUs per horse in future studies may provide a more robust understanding of normative amounts of Chuckwagon racehorse asymmetry going forward.

### 4.2. Normal Asymmetries May Be Unique in Chuckwagon Racing

Our current study provides the first quantitative evidence for Chuckwagon racehorse asymmetries. Movement asymmetries are commonly observed in other racing disciplines. Thoroughbreds often present with asymmetries that can vary over days or weeks [16,18] and are associated with consistently training and racing in one direction [17]. Several studies have suggested normal asymmetry thresholds in riding horses and some racing disciplines [19,27,28,29]. After comparing IMU gait analysis with expert visual lameness scores, our group suggested a preliminary guideline of ≥14.5 mm (based on MnD values) for forelimb lameness in Thoroughbred racehorses [19]. On average, the Chuckwagon racehorses in the present study were within this threshold for weight-bearing asymmetry, which reflects MnD values. However, seven weight-bearing measures from seven uninjured horses were above the threshold in warmups, and six measures from five uninjured horses were above the threshold in cooldowns. The injured horse in our study did not present with weight-bearing values above this threshold. Further work should investigate how weight-bearing asymmetries in Chuckwagon racehorses may differ from other disciplines.

Macaire et al. (2022) suggested lameness thresholds of 10% ROM for upwards movement asymmetry measured at the withers in riding horses [27]. It is worth noting that our measures were taken from the harness at the midline in the cranial/mid thoracic area as this is a convenient placement of the sensor onto existing parts of the Chuckwagon harness equipment. Therefore, these thresholds may not be directly comparable, but they provide a baseline to begin understanding how normal Chuckwagon racehorse push-off asymmetries may differ from other disciplines. On average, our push-off asymmetry values, measured from a harness-mounted sensor slightly caudal to the withers, were well above this threshold both before and after racing. There were 61 measures from 45 horses above the threshold in warmup, and 70 measures from 47 horses above the threshold in cooldown, including all measures from the injured horse. Considering we found significantly increased % ROM push-off asymmetries after racing, this suggests that push-off asymmetries may be of particular interest for future investigations into MSK injuries in Chuckwagon racing.

### 4.3. Moving Towards Assessing Chuckwagon Racehorse Injuries Going Forward

This study presents data from one horse that sustained an MSK injury, specifically a catastrophic fracture to the left forelimb cannon bone during racing. Our injury data is limited to one horse and cannot conclusively assess injuries in relation to asymmetry measures in this population, but the data allows for a brief insight into how Chuckwagon MSK injuries may be further addressed in future studies.

Drivers had the choice of which horse participated on any given race day, so by chance this horse was instrumented with a sensor 4 days, 3 days, and 1 day before the injury, and on the day of injury. Apart from warmup values 4 days prior to injury, most movement asymmetry values for this horse exceeded the average of the uninjured horses. Several weight-bearing, and all push-off measures for this individual exceeded suggested lameness thresholds of 10% ROM for withers movement [27]. There appears to be a progressive increase from 4 days before the injury to 3 days, but whether this pattern relates to the subsequent injury cannot be determined conclusively without more information.

Importantly, many uninjured horses in the sample had higher weight-bearing asymmetries, and a more limited number had higher push-off asymmetries. The overlap between injured and uninjured values may complicate any future injury models in this unique discipline. Future work should aim to determine whether other factors may explain the underlying reasons for the observed increase in % ROM push-off asymmetries observed here. For example, Persson-Sjodin et al. (2019) showed that riding horses with movement asymmetries may not show significant differences between medicated pain treatment and placebo [30], rendering a direct link between asymmetry and pain difficult to prove. In a follow-up study, horses with clinical lameness responded to local anesthesia, suggesting pain was the cause of asymmetry in these horses [31]. In general, fundamental principles combined with clinical evidence in lame horses suggest a direct link between movement symmetry changes and force distribution between contralateral limbs [32,33,34]. The relationships between normal variation, pain, injuries, and movement symmetry are complex, and should be further investigated in Chuckwagon racehorses to better understand which movement patterns might accurately predict injuries. Further, movement symmetry is just one of many locomotor characteristics that may need to be considered in the context of potential future injury predictions for this unique discipline.

Building a longitudinal evidence base may allow stakeholders to continuously modify or implement new policies that further protect these racehorses from excessive loading and injuries. Many catastrophic injuries are chronic in nature [2] and there are other longer-term factors that we did not measure, but which could be important when considering injury risk. These include how many times a horse races in a season [35] and non-catastrophic injuries that may have occurred throughout the season. Some of the horses in this sample may perform more races in the season than others, and some may have sustained injuries after the Calgary Stampede that we are unaware of. Overall, more robust evidence bases are needed to better understand Chuckwagon racehorse MSK injury risk going forward.

Wearable sensors provide a practical tool to monitor asymmetry over several days of racing. Other groups have used similar methodologies to assess locomotion in other race disciplines [36,37,38,39]. Importantly, the methods used here did not require the horses to undergo extra exercises such as in-hand trot-ups. Our approach is suitable for Chuckwagon racing as sensors can be attached to harnesses, and horses commonly trot during warmups and cooldowns. However, not all individuals trot during these exercises, and their self-selected gait can be influenced by the driver or surrounding horses. Despite these limitations, GNSS-IMU devices can be used as non-disruptive tools for continuous monitoring of symmetry and other locomotor characteristics in dynamic racing settings. Overall, wearable sensors may provide an opportunity for more proactive racehorse MSK injury management based on changing movement patterns over time in the future.

### 4.4. Limitations and Future Directions

It should be noted that at a sampling rate of 25 Hz, which was the sample rate of the sensors used here, root mean square errors in comparison to measurements at 100 Hz sample rate are in the order of from 3 to 7 mm [40]. This renders interpretation of small individual changes observed before and after racing with the current setup difficult. Other devices that combine GPS or GNSS data at lower (10 to 25 Hz) sample rate with IMU data captured at higher sample rate (50 Hz or above) would offer a higher precision for IMU measurements while also allowing for calculation of in-race gallop stride parameters. Therefore, the sampling rate of devices should be considered before any confident conclusions can be drawn from an individual racehorse’s locomotor changes in future work.

The preliminary nature of this study with only ten GNSS-IMU sensors available did not allow us to confidently assess the influence of horse position (left vs. right leader or wheeler), driver, or differences across days. We did check these factors by adding them to our models and did not observe any differences, so we cannot conclusively identify whether they may influence the relationship between asymmetries before and after racing without more comprehensive datasets. More robust quantitative evidence from longitudinal studies where multiple horses per outfit are regularly instrumented are needed to assess these relationships. This would also allow for assessment of short-, medium-, and long-term asymmetry patterns over an entire race season.

Most of our linear models found that between-horse variability accounted for approximately 80% of the total variance, indicating that these movement metrics are highly individual-specific. This suggests that deviations from an individual horse’s baseline, rather than group-level thresholds, may be more appropriate for identifying meaningful changes in future work that aims to monitor Chuckwagon racehorse movement over time. Work by Sepulveda Caviedes et al. (2018) indicates that day-to-day and weekly variability in movement symmetry is high in Thoroughbreds in training [18], which may also partly explain why we did not observe many significant relationships. More controlled longitudinal datasets may allow us to explore asymmetries in Chuckwagon racehorses in more detail to help determine their potential relevance for future injury prediction work.

Age should also be considered in future Chuckwagon research. Many Chuckwagon racehorses previously competed in flat racing, typically from ages 2–5, then continue their careers in Chuckwagon racing until their late teens. Because of the preliminary nature of the present study and limited time available to interact with the drivers at the high-pressure environment at the Calgary Stampede Rangeland Derby, we had a limited amount of time to collect additional information about the horses. This study was designed not to interfere with racing practices, including not interfering with barn routines, such as vet visits that drivers and their teams participate in prior to racing each day. That being said, older age has been related to injury risk in other racing disciplines [41], so future studies that aim towards building MSK injury prediction models in this specific racing discipline should include age in their analyses.

This study also did not take the surface characteristics of the racetrack into account. Surface characteristics of a dirt track can influence stride characteristics in Chuckwagon outrider horses [21], so the influence of surface on movement symmetry in wagon horses should be investigated to better understand normal levels of asymmetries in this discipline. Together with health, performance, and injury-related information, longitudinal studies may allow us to establish normative levels of asymmetry before and after Chuckwagon races and assess whether this information is important for injury prevention.

## 5. Conclusions

Trot measures obtained from GNSS-IMU sensors attached to Chuckwagon racehorse harnesses showed small increases in push-off asymmetry during post-race cooldowns when normalized as percent range of motion (% ROM). This may be related to the accumulation of in-race MSK loads. One horse with high levels of movement asymmetry measured over multiple days sustained a catastrophic MSK injury during racing. However, other horses with elevated asymmetries compared to the injured horse or suggested thresholds from other Thoroughbred racehorse disciplines did not sustain injuries. Longitudinal work is needed to establish normal levels of asymmetry before and after Chuckwagon racing, as well as other factors that may influence injury risk, before we can attempt to assess whether asymmetry values can be used to predict injuries similarly to other racing disciplines [36,38,39]. GNSS-IMU devices offer practical, non-disruptive tools to assess movement symmetry in dynamic racing settings and may present a possible avenue for proactive racehorse movement monitoring and injury risk management.

## Figures and Tables

**Table 1:** Number of trotting measures obtained per day. The distribution of measurements is not identical on each day as there were differences in horse availability on any given day, and not all horses select a trotting gait at each warmup and cooldown on each day.

Days	Total (*n* = 156)	Warmup (*n* = 73)	Cooldown (*n* = 83)
Day 1	8	4	4
Day 2	13	6	7
Day 3	18	9	9
Day 4	16	7	9
Day 5	18	9	9
Day 6	18	9	9
Day 7	9	3	6
Day 8	18	8	10
Day 9	17	9	8
Day 10	15	6	9

## Data Availability

The data presented in the study are openly available in Figshare at [https://doi.org/10.6084/m9.figshare.31272238].

## Abbreviations

The following abbreviations are used in this manuscript:

UpD	Upward difference
MnD	Minimum difference
ROM	Range of motion
Amp1	First amplitude of upward movement in a stride cycle
Amp2	Second amplitude of upward movement in a stride cycle
LF	Left front
RF	Right front
GNSS-IMU	Global Navigation Satellite Systems with Inertial Measurement Units
LL	Left leader
RL	Right leader
LW	Left wheeler
RW	Right wheeler
